# Histamine Potentiates SARS-CoV-2 Spike Protein Entry Into Endothelial Cells

**DOI:** 10.3389/fphar.2022.872736

**Published:** 2022-04-25

**Authors:** Somasundaram Raghavan, M. Dennis Leo

**Affiliations:** Department of Pharmaceutical Sciences, University of Tennessee Health Science Center, Memphis, TN, United States

**Keywords:** SARS-CoV-2, histamine, famotidine, spike, angiotensin-converting enzyme-2

## Abstract

Severe acute respiratory syndrome coronavirus 2 (SARS-CoV-2) which causes coronavirus disease (COVID-19) is one of the most serious global health crises in recent history. COVID-19 patient symptoms range from life-threatening to mild and asymptomatic, which presents unique problems in identifying, quarantining, and treating the affected individuals. The emergence of unusual symptoms among survivors, now referred to as “Long COVID”, is concerning, especially since much about the condition and the treatment of it is still relatively unknown. Evidence so far also suggests that some of these symptoms can be attributed to vascular inflammation. Although famotidine, the commonly used histamine H2 receptor (H2R) blocker, was shown to have no antiviral activity, recent reports indicate that it could prevent adverse outcomes in COVID-19 patients. Histamine is a classic proinflammatory mediator, the levels of which increase along with other cytokines during COVID-19 infection. Histamine activates H2R signaling, while famotidine specifically blocks H2R activation. Investigating the effects of recombinant SARS-CoV-2 spike protein S1 Receptor-Binding Domain (Spike) on ACE2 expression in cultured human coronary artery endothelial cells, we found that the presence of histamine potentiated spike-mediated ACE2 internalization into endothelial cells. This effect was blocked by famotidine, protein kinase A inhibition, or by H2 receptor protein knockdown. Together, these results indicate that histamine and histamine receptor signaling is likely essential for spike protein to induce ACE2 internalization in endothelial cells and cause endothelial dysfunction and that this effect can be blocked by the H2R blocker, famotidine.

## Introduction

Severe acute respiratory syndrome coronavirus 2 (SARS-CoV-2) virus, which causes coronavirus disease (COVID-19), has swept across the globe over the last 2 years, inflicting enormous damage on both personal health and societal well-being (Ackermann et al., 2020; Wiersinga et al., 2020; Hu et al., 2021; Nalbandian et al., 2021). While most patients are either asymptomatic or show mild-to-moderate symptoms, around 15% of affected individuals might require hospitalization and ∼ 5% might need intensive care (Nalbandian et al., 2021). While several studies that have been carried out so far have broadened our understanding of the acute phase of the disease, it has become clear that many individuals who had been infected but recovered have developed mysterious and wide-ranging health issues. These varied symptoms are now collectively referred to as “Long COVID” and include several symptoms that suggest vascular inflammation and dysfunction (Ackermann et al., 2020; Marshall, 2020; Maxwell et al., 2021; Varga et al., 2020; Vassiliou et al., 2020).

The “cytokine storm” that occurs in acute-COVID-19 infection is associated with a massive surge in the circulating levels of several proinflammatory cytokines, but the relevance of histamine, a classic proinflammatory mediator, has been controversial (Ghosh et al., 2020; Loffredo et al., 2021; Ennis and Tiligada, 2021; Malone et al., 2021). Mast cells infected with SARS-CoV-2 release histamine (Conti et al., 2020), while famotidine, a histamine receptor-2 (H2R) blocker, itself does not inhibit virus replication (Conti et al., 2020), and it has been suggested that it might have potentially beneficial effects over the course of the disease (Malone et al., 2021).

ACE2 is a type I membrane-localized glycoprotein that is highly expressed in several cell types and is the primary receptor used by SARS-CoV-2 for cellular entry (Walls et al., 2020). While present in abundance in the heart, conflicting reports have emerged on its expression in vascular cells, specifically endothelial cells, and if the virus can directly infect endothelial cells. Previous evidence had revealed that the virus did not readily infect endothelial cells *in vitro* requiring ACE2 overexpression (Nascimento Conde et al., 2020), while another study reported that there were differences in viral entry into the endothelial cells from different human arteries (Wagner et al., 2021).

The SARS-CoV-Spike (S, spike) protein, which binds ACE2, is a structural protein comprising S1 and S2 subunits with the “Receptor Binding Domain” in the S1 subunit (Zhang H. et al., 2020; Huang et al., 2020). We had also shown previously that the recombinant spike protein added alone to normal cultured endothelial cells required ∼ 12 h for observable downregulation of endothelial barrier function (Raghavan et al., 2021). Based on the available data so far, we hypothesized that histamine signaling through the endothelial H2 receptors could accelerate spike-induced ACE2-internalization in the endothelial cells to then trigger endothelial dysfunction. Our results here show that in healthy, untreated, and primary human coronary artery endothelial cells, the addition of the recombinant spike protein did not induce ACE2 internalization immediately but took around 6–12 h for internalization and degradation. The addition of histamine to endothelial cells accelerated spike-ACE2 internalization with observable intracellular ACE2 protein within 30 min of treatment. Blocking the H2R signaling pathway with either famotidine, the protein kinase A inhibitor, PKI, or knockdown of histamine receptor (H2R) inhibited spike-induced ACE2 internalization. Thus, these results suggest that histamine might be an important cofactor for internalization of the ACE2 and likely plays a significant role in accelerating spike protein entry into endothelial cells.

## Materials and Methods

### Cell culture

The human primary coronary artery endothelial cells were purchased from Cell Biologics Inc. The cells were cultured in the endothelial cell medium containing (0.5 ml VEGF, Heparin, EGF, FGF, Hydrocortisone, L-Glutamine, and Antibiotic–Antimycotic Solution) supplemented with 2% fetal bovine serum.

### Reagents

The SARS-CoV-2 (COVID-19) S1 Recombinant Protein (cat. no. 97-092) was purchased from ProSci Inc. (Poway, CA) and used at a final concentration of 10 μg/ml. The endothelial cells were treated with spike protein at the final concentration for the required time before surface biotinylation experiments were carried out. The ACE2 antibody was purchased from Abnova (Taipei, Taiwan, cat. no. PAB13443). Histamine, famotidine, and PKI were purchased from Sigma-Aldrich (St. Louis, MO, United States). siRNA to H2 receptor and scrambled siRNA were purchased from Thermo Fisher Scientific Inc.

### Surface Biotinylation of Intact Endothelial Cells

Surface biotinylation was performed as we have done previously for intact arteries with slight modifications (Leo et al., 2017; Leo et al., 2018; Hasan et al., 2019; Leo et al., 2021). Briefly, live endothelial cell cultures were treated with appropriate reagents for specified times. After the treatment period, the reagents were washed with warm phosphate-buffered saline (PBS), and the cell culture plates were immediately placed on ice to inhibit all protein trafficking. The cell-impermeable biotinylation reagents dissolved in PBS are then added to the cultures and allowed to incubate at 4°C with gentle shaking for 30 min. The biotinylation reagents are removed with a PBS wash, and the reaction is quenched with 100 mM glycine. Protein lysates are prepared from each culture, and protein content is estimated. An equal quantity of protein from each group is passed through an avidin bead column to separate the biotinylated surface protein, which is eluted from the beads and prepared as the “surface fraction.” The unbound protein was collected as the intracellular fraction. Each sample was then run as contiguous lanes on an SDS-gel as surface (denoted as ‘S’) and intracellular (denoted as ‘I’) fractions. The analysis was carried out by semi-quantitative comparison between the surface band intensities compared to the untreated or scrambled siRNA controls and was expressed as the ACE2 surface protein, % untreated control.

### Western Blotting

Western blotting for protein was carried out following the standard protocols. After separation of surface and intracellular fractions, the proteins were separated on 7.5% SDS-polyacrylamide gels and transferred onto nitrocellulose membranes. The membranes were blocked with 5% non-fat milk and incubated with the ACE2 antibody overnight at 4°C. The membranes were washed and incubated with the horseradish peroxidase–conjugated secondary antibodies at room temperature. The blots were physically cut to allow for probing of two different proteins without the need for stripping. The protein bands were imaged using a ChemiDoc gel imaging system and quantified using Quantity One software (Biorad).

### Statistics

Statistical analysis was performed using OriginLab and GraphPad InStat software. The data are shown as the mean ± SE and expressed as % change compared to the untreated surface ACE2 band intensities. Student’s t-test and Mann–Whitney U test were implemented, where appropriate *p* < 0.05 was considered statistically significant.

## Results

### Recombinant Spike Protein Does Not Readily Induce Endothelial Cell ACE2 Internalization

**FIGURE 1:**
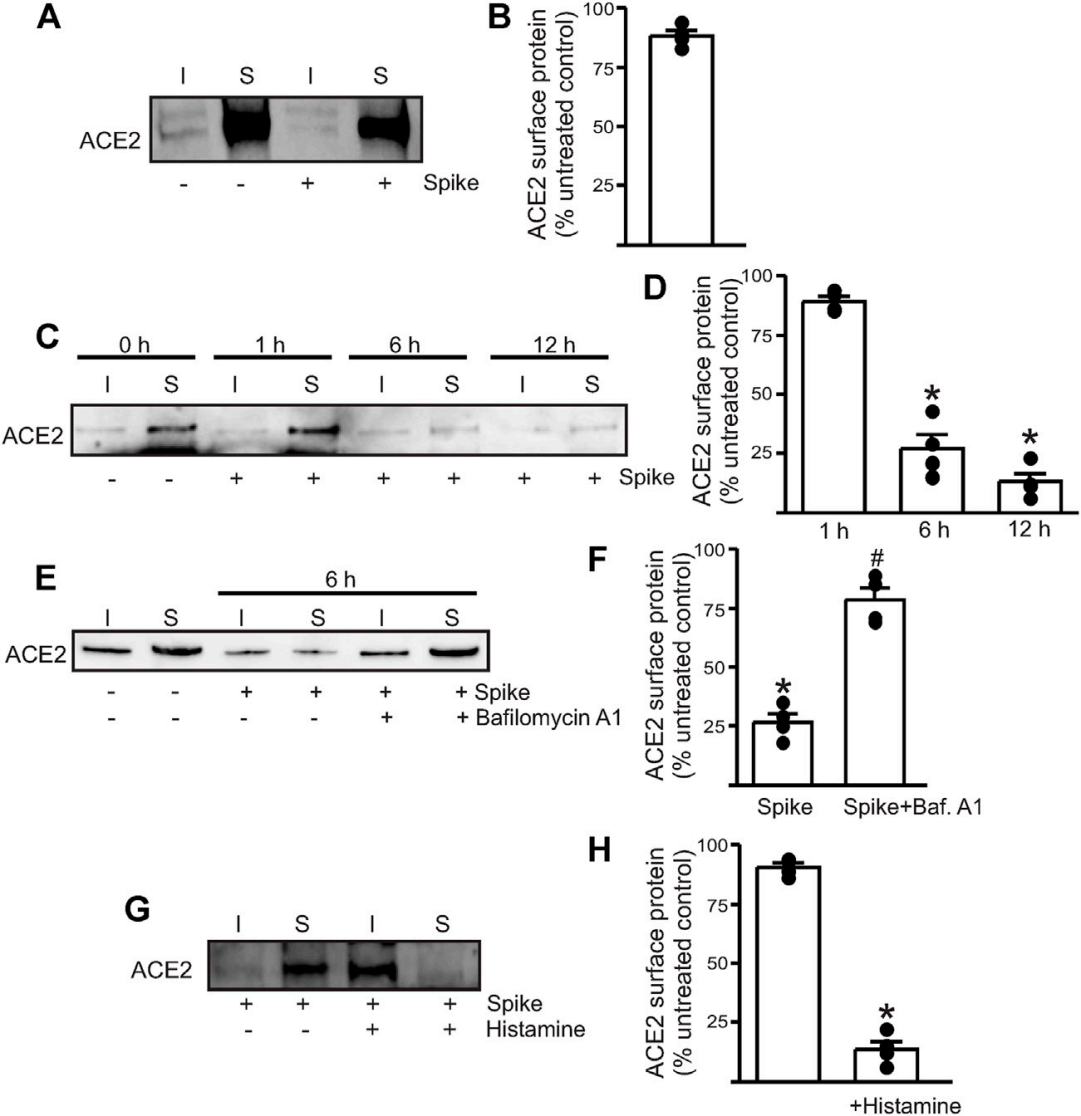
Spike-induced ACE2 internalization is enhanced in the presence of histamine. **(A)** Representative Western blotting after surface biotinylation of intact endothelial cells with or without spike treatment for 30 min. **(B)** Mean data. **(C)** Representative Western blot after surface biotinylation showing that increased incubation time after spike treatment induced ACE2 internalization and degradation. **(D)** Mean data. *p < 0.05 *vs*. surface band intensity of the untreated control. **(E)** Representative Western blot after surface biotinylation showing the effect of bafilomycin A1 on spike-induced ACE2 degradation. **(F)** Mean data, *p < 0.05 *vs*. untreated, #*p* < 0.05 *vs*. spike treated. **(G)** Representative Western blot after surface biotinylation showing that histamine potentiates spike-induced ACE2 internalization within 30 min of treatment. **(H)** Mean data, *p < 0.05 *vs*. surface band intensity of untreated control. *n* = 4 for all the experimental sets. I- indicates intracellular fraction, S-denotes surface or plasma membrane fraction.

To determine if the spike can induce endothelial cell ACE2 internalization, we performed surface biotinylation of live endothelial cells. Recombinant spike (10 μg/ml) was added to a confluent plate of endothelial cells and allowed to incubate for 30 min. After this time, warm PBS was used to replace the media, and then the biotinylation reagents dissolved in PBS were added to the cells, and surface biotinylation was performed for 30 min at room temperature. First, the results showed that ACE2 is predominantly a plasma membrane (surface)–localized protein in healthy endothelial cells with >85% of the protein located on the surface (Figures 1A,B) Second, the addition of the spike protein alone to normal, healthy endothelial cells did not induce any ACE2 internalization at 30 min (Figures 1A,B). Previously, we had shown that the spike protein induced endothelial junctional protein degradation after 12-h treatment of mouse endothelial cells (Raghavan et al., 2021). Hence, we next tested if longer incubation times would induce ACE2 internalization and degradation. The results showed that spike treatment for 6 h reduced the ACE2 total and surface expression by ∼ 70% compared to that of the untreated controls and by ∼ 85% after 12 h of spike treatment (Figures 1C,D). In separate experiments, the endothelial cells were first pretreated for 30 min with bafilomycin A1, a lysosomal H^+^-ATPase inhibitor, and then treated with the spike protein for 6 h in the presence of the inhibitor. The results show that bafilomycin A1 rescued ACE2 protein from spike-induced degradation which was then retrafficked to the cell surface (Figures 1E,F). This suggests that internalized ACE2 undergoes degradation *via* the lysosomal pathway. Taken together, these results indicate that in the healthy endothelial cells, the presence of the spike protein alone does not induce immediate internalization of the ACE2 receptor. Prolonged exposure to spike protein induces ACE2 internalization. The results also show that the internalized ACE2 is degraded and is not stored intracellularly.

### Histamine Accelerates Spike Protein–Induced ACE2 Internalization

Since histamine levels are likely to increase during COVID-19 infection, we next tested if the presence of histamine modified the interaction of spike with ACE2. The control endothelial cells were treated with 1 µM histamine along with the spike protein for 30 min after which the reagents were washed off with warm PBS and surface biotinylation was performed at 4°C. Western blotting showed that in the presence of histamine, almost all the surface ACE2 now appeared in the intracellular fraction (Figures 1G,H). Given the very brief incubation time with the spike, this result indicates that 1. histamine significantly potentiates the ability of the spike to internalize ACE2 and 2. ACE2 is first internalized into the cell before undergoing degradation, and it is likely that a longer incubation period or sustained spike presence is required for ACE2 to undergo degradation. Overall, this suggests that histamine plays an important role in mediating spike–ACE2 interaction on the cell surface.

### Histamine Acts Through Endothelial H2 Receptors to Accelerate Spike–ACE2 Internalization

**FIGURE 2:**
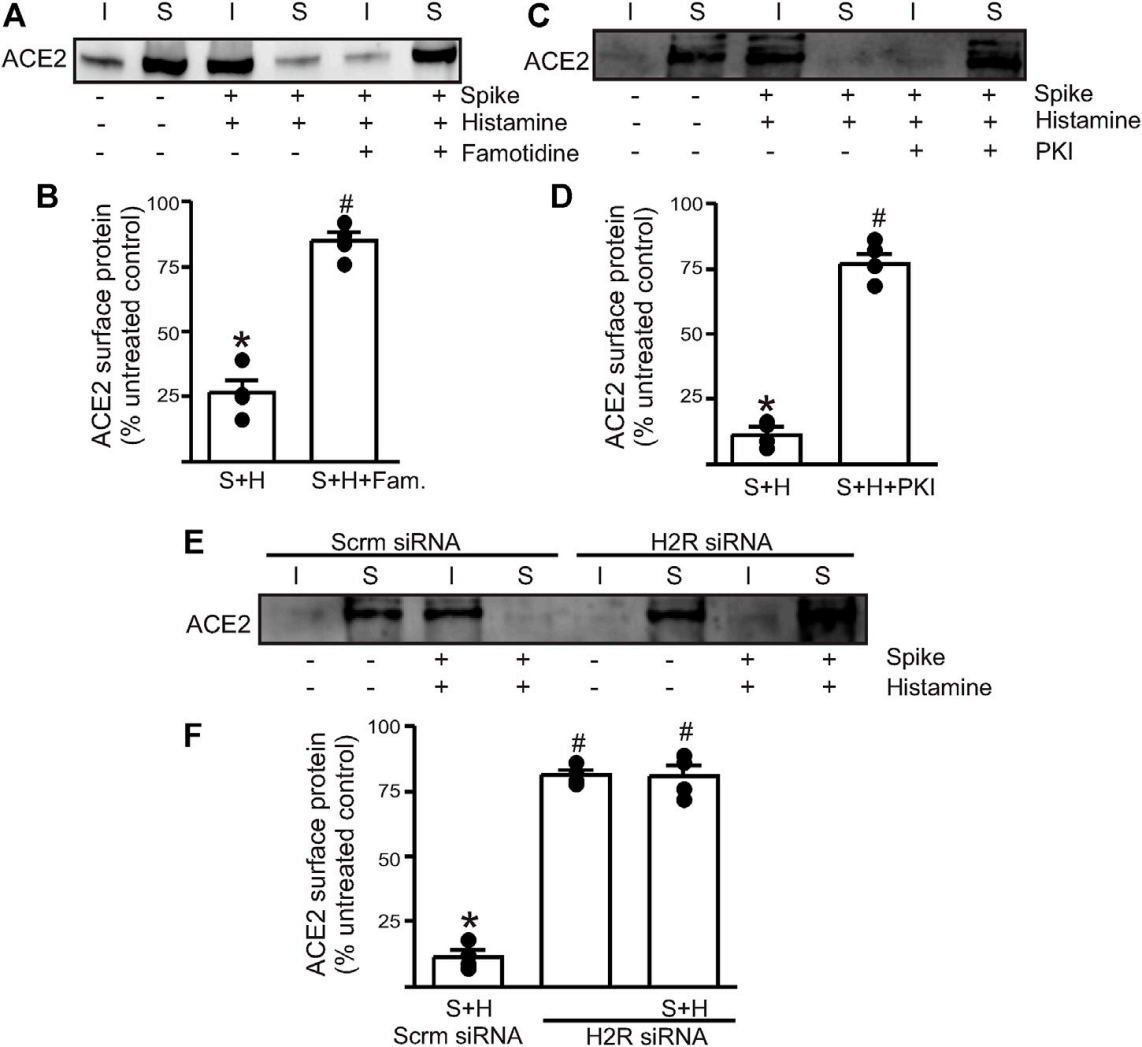
Histamine H2 receptor signaling is involved in histamine potentiating spike–ACE2 internalization. **(A)** Representative Western blot after surface biotinylation of the intact endothelial cells showing the effect of famotidine on the potentiating effect of histamine on spike-ACE2 internalization. **(B)** Mean data. **(C)** Representative Western blot after surface biotinylation showing the effect of the protein kinase A inhibitor, PKI, in preventing histamine-induced spike–ACE2 internalization. **(D)** Mean data. *p < 0.05 *vs*. the untreated control, #*p* < 0.05 *vs*. spike + histamine. **(E)** Representative Western blot after surface biotinylation showing the effect of H2 receptor protein knockdown on spike + histamine treatment. Scrm-scrambled siRNA. **(F)** Mean data. *p < 0.05 *vs*. untreated control, #*p* < 0.05 *vs*. spike + histamine scrambled control. *n* = 4 for all the experimental sets. I- indicates intracellular fraction, S-denotes surface or plasma membrane fraction.

Next, we wanted to investigate which endothelial signaling pathways were activated in the presence of histamine. We hypothesized that histamine likely acted through the endothelial H2 receptors to increase spike–ACE2 internalization. Accordingly, we pretreated the endothelial cells with famotidine (10 µM), the specific H2R blocker, for 30 min before the addition of spike and histamine. This combination was kept for an additional 30 min after which they were washed from the cell culture dishes with warm PBS and surface biotinylation was performed at 4°C. The results showed that in the presence of famotidine, much of the ACE2 protein was still present at the cell surface after treatment with spike and histamine (Figures 2A,B). These data suggest that famotidine, likely by the inhibition of H2R and its downstream signaling, prevented histamine from accelerating the internalization of ACE2.

To further investigate the involvement of H2R, we tried two different approaches. First, we pretreated the endothelial cells with PKI (10 µM), a protein kinase A (PKA) inhibitor. Histamine stimulates H2R to activate downstream PKA signaling, so the blockade of PKA signaling should negate H2R activation. The results showed that pretreatment of endothelial cells with PKI before the addition of spike + histamine completely prevented spike–ACE2 internalization (Figures 2C,D), suggesting that downstream PKA signaling after H2R activation was essential to this process. Second, we transfected the control cells with siRNA to H2R to knockdown the H2R protein. The results indicated that in cells where H2R was knocked down, the spike + histamine combination did not affect ACE2 surface expression compared to the scrambled siRNA controls (Figures 2E,F).

Together, these data indicate that extracellular histamine *via* the activation of H2R signaling potentiates spike-induced ACE2 internalization and that the inhibition of H2R by famotidine prevents acute internalization of the cell surface–localized ACE2 protein.

## Discussion

Here, we investigated the hypothesis that SARS-CoV-2 spike protein-induced ACE2 internalization is accelerated in the presence of histamine and that famotidine, a commonly used histamine receptor blocker, can block endothelial cell–spike protein entry through the ACE2 receptor. Using surface biotinylation to track the cellular localization of ACE2 in the endothelial cells after recombinant spike protein treatment, our data here showed that in the normal human coronary artery endothelial cells, the spike protein alone had a delayed response in internalizing ACE2. The total and surface ACE2 were reduced by ∼ 85% only after 12 h of incubation with spike. In contrast, in the presence of histamine, the spike induced ACE2 internalization within 30°min. This effect was blocked when the endothelial cells were pretreated with famotidine, the PKA inhibitor, PKI, or when the H2 receptor was knocked down using siRNA. These results showed that the presence of histamine, acting through the endothelial H2 receptors, potentiated endothelial ACE2 internalization.

Evidence of vascular damage in COVID-19 patients began to emerge even early during the pandemic (Gavriilaki et al., 2020; Siddiqi et al., 2021), but there are contrasting reports on the virus infection of vascular cells, specifically endothelial cells. In opposition to the hypothesis that the virus directly infects endothelial cells, Nascimento Conde et al. (2020) have shown that recombinant ACE2 was required for the virus to infect the endothelial cells (Nascimento Conde et al., 2020), while other authors have argued that the expression of ACE2 in the endothelial cells might be too low to support viral entry (McCracken et al., 2021). In contrast, several other groups have found that human endothelial cells do express ACE2 and that the virus likely uses several additional protein pathways to enter the endothelial cells (Hamming et al., 2004; Zhang J. et al., 2020; Zhao et al., 2020; Albini et al., 2021; Wagner et al., 2021; Zhang et al., 2021; Amraei et al., 2022). In an earlier study, we too have shown that the recombinant spike protein incubated with mouse cerebral endothelial cells for 12 h not only induced ACE2 degradation but also decreased endothelial junctional protein expression, thereby affecting endothelial barrier functionality (Raghavan et al., 2021). Here, we also show that inhibition of the lysosomal degradative pathway rescued the internalized ACE2 from degradation. These results were corroborated in mouse models, where intravenous injections of the spike protein resulted in the protein appearing in several regions of the brain, likely after blood–brain barrier disruption and was found to cause cerebral endothelial microvascular damage and that the lysosomal pathway was involved in the virus uncoating within the cell [(Ballout et al., 2020), (Blaess et al., 2020), (Davenport et al., 2021), (Nuovo et al., 2021), (Rhea et al., 2021), (Shang et al., 2020)]. These contrasting reports not only highlight the complexity of COVID-19 but also suggest that ACE2 expression in the vasculature could be highly variable, thus requiring further investigation.

Famotidine (*Pepcid®*) is a histamine H2 receptor–selective blocker, approved by the FDA for treatment of gastroesophageal reflux disease (GERD) and gastric ulcer. Early in the pandemic, evidence emerged that the hospitalized COVID-19 patients treated with famotidine had a reduced risk of severe outcome and/or mortality (Freedberg et al., 2020; Mather et al., 2020; Sethia et al., 2020). Several groups have hypothesized that this effect of famotidine was likely due to the direct inhibition of the histamine H2 receptor (H2R) rather than antiviral activity (Ghosh et al., 2020; Loffredo et al., 2021; Ennis and Tiligada, 2021). Indeed, famotidine was later shown to have no SARS-CoV-2 antiviral activity (Malone et al., 2021), while conflicting reports on its effectiveness in acute COVID-19 cases and the availability of better COVID-19 treatments saw the drug gradually fall out of favor for this purpose. However, the uptick in abnormal health issues in some COVID-19 survivors, which is now broadly described under the term “Long COVID” and the possibility that some of these symptoms might be associated with the long-term virus-induced inflammatory damage to the vasculature, has re-ignited interest in this drug (Eldanasory et al., 2020; Malone et al., 2021; Mura et al., 2021). A recent outcome study of more than 250,000 COVID-19 infected patients found that the histamine receptor blockade significantly improved the outcomes in patients who needed respiratory support and has suggested that this might be due to the ability of famotidine to block the proinflammatory signals arising from H2R activation (Mura et al., 2021).

Our brief investigation here has shown that the presence of histamine accelerates the spike protein–induced ACE2 internalization in the endothelial cells. In the absence of histamine, the spike protein alone required between 6 and 12 h for the ACE2 to be internalized and degraded. However, in the presence of histamine, the ACE2 was internalized within 30 min. In addition, this effect was largely blocked by either the knockdown of H2R, inhibition of PKA, the effector of the H2R signaling pathway, and most importantly, by famotidine, or the well-known H2R blocker. These results suggest that histamine and the histamine receptor signaling pathway might play a crucial role in virus entry into the endothelial cells. SARS-CoV-2 elicits an inflammatory reaction in the infected patients, leading to the production of several proinflammatory substances including histamine (Nalbandian et al., 2021). Several of these mediators such as TNF-α and several interleukins can further stimulate inflammatory cells such as mast cells to increase the synthesis and secretion of histamine (Crook et al., 2021; Nalbandian et al., 2021; Raveendran et al., 2021). Similar to what was observed with histamine here, the circulating vimentin has also been shown to facilitate SARS-CoV-2 endothelial cell entry (Amraei et al., 2022). Ours and other such studies suggest that the circulating or local levels of endogenous substances could potentiate the entry of the virus into the endothelial cells. We acknowledge the obvious limitation of this study, which is the concentration of histamine used to treat the cells (1 µM). While it is not likely that this concentration would be achieved in circulation in humans, the possibility remains that the local elevations in histamine levels, for example, in the lung, brain, or the heart could alter the viral entry into the cells in the immediate vicinity. We also utilized the recombinant spike protein of the original SARS-CoV-2 variant. Hence, it will be interesting to study the effects of the highly transmissible variants that appeared later and if the spike proteins from these variants could circumvent the requirement of the circulating cofactors such as vimentin or histamine.

## Conclusion

COVID-19 is a complex disease and while vaccinations and better treatments options have effectively limited the morbidity and mortality, much remains to be learned about ‘Long COVID’ and its treatment. While famotidine does not have SARS-CoV-2 antiviral activity, the data presented here suggest that famotidine could be considered a second-line treatment to limit virus-induced vascular inflammation.

## Data Availability

The original contributions presented in the study are included in the article/Supplementary Material, further inquiries can be directed to the corresponding author.
