# Supplementary figures and images for: Histamine Potentiates SARS-CoV-2 Spike Protein Entry Into Endothelial Cells
Source: Front Pharmacol. 2022 Apr 25;13:872736. doi: 10.3389/fphar.2022.872736 (PMC9084361; doi:10.3389/fphar.2022.872736)

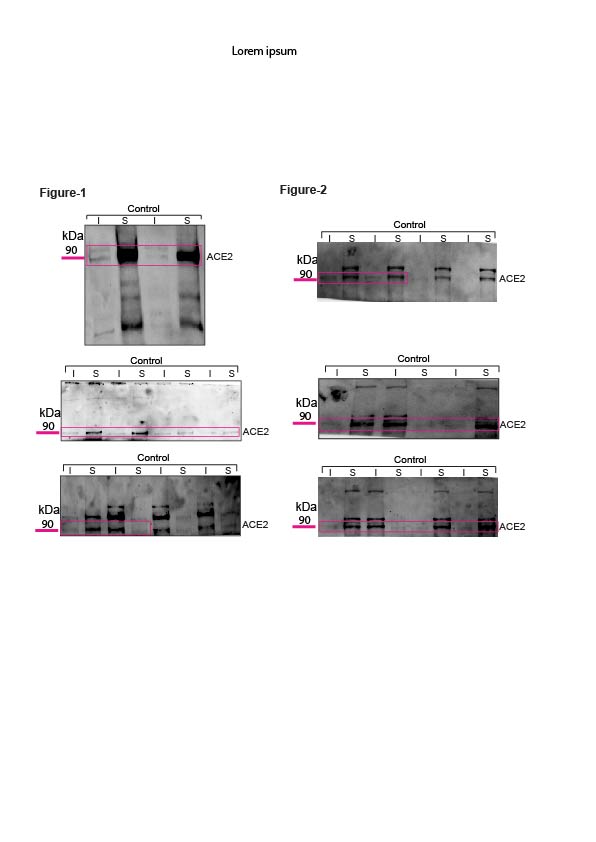

Supplement: Supplementary file 1 [file Image1.JPEG]
